# Temporal dynamics of prognostic factors in breast cancer survival

**DOI:** 10.1371/journal.pone.0349115

**Published:** 2026-05-27

**Authors:** Ingunn Fride Tvete, Marianne Klemp

**Affiliations:** 1 Norwegian Computing Center, Oslo, Norway; 2 The University of Oslo, Department of Pharmacology, Oslo, Norway; Örebro University Faculty of Medicine and Health: Orebro universitet Fakulteten for medicin och halsa, SWEDEN

## Abstract

**Objectives:**

We aimed to explore how relevant factors could have shifting prognostic impacts on mortality among BC patients over time, applying alternative survival model specifications.

**Methods:**

Given data from the Cancer Registry of Norway we followed 36 412 women aged 40 + , breast cancer (BC) diagnosed 2006–2020. We analyzed survival comparing patient’s molecular subtype group and BMI, adjusting for age, tumor stage and treatment, considering incidence rates for BC specific death (BCSD) and other causes of death (OCOD). We explored cause-specific survival models with and without time invariant coefficients.

**Results:**

Altogether 8.6% (2 542 patients) and 7.0% (3 125 patients) died from other causes and BC throughout the study period, respectively.Molecular subtype HR + /HER2-patients had similar BCSD and OCOD incidence rates. For the other patients the BCSD incidence was higher than the OCOD incidence, especially for HR-/HER2+ and HR-/HER2- patients. Allowing for the association between time to BCSD and molecular subtype groups and radiation therapy to vary over time we found that HR-/HER2+ and HR-/HER2- patients had hazard ratios of 3.38 and 3.53 compared to HR + /HER2- patients within 3 years following BC diagnosis, and hazard ratios of respectively 0.93 and 1.62 in the subsequent years. The positive effect of radiation therapy on BCSD was highest in the first years following diagnosis and still positive, albeit much smaller, in the later years. We found no difference in BC mortality risk between underweight, normal weight, overweight and obese patients.

**Conclusions:**

Our findings contribute to understanding long-term BC survival. Cause-specific Cox models that assume proportional hazards do not allow for time-varying coefficients. This may lead to overlooked time-varying effects of relevant prognostic factors. Especially with longer time horizons one might draw incorrect conclusions.

## Introduction

Survival rates for breast cancer (BC) patients have improved over years due to earlier detection and better treatment [1]. However, the incidence of BC is increasing worldwide [2–3]. E.g., BC incidence in Norway for women was 139.6 per 100 000 in 2024 [4]. With increasing incidence and survival, the BC surviving population will increase, making it more relevant than ever to describe long-term survival paths given relevant patient risk factors and treatment. Major variation in BC survival has been described [5], and patients’ prognoses depend largely on the molecular subtype, in addition to factors such as age, tumor stage and treatment. In this paper we explore how these factors could have shifting prognostic impacts on mortality among BC patients over time, applying alternative survival model specifications. We study time to breast cancer specific death (BCSD) with other causes of death (OCOD) as a competing risk, comparing patient’s molecular subtype groups and BMI levels, given patient characteristics (age and tumor stage) and radiation therapy. We consider three alternatives for specifying the association between time to BCSD and patient characteristics and radiation therapy treatment over the years where we assume:

1. a time-invariant association over the years,
2. a time-varying association described by splines and
3. different associations for different time intervals for some factors (set by considering the associations found in 2).

That the association between time to BCSD and factors can vary over longer time horizons is not new knowledge [6,7]. This study illustrates how different model specifications provide insight into this variation and is an important reminder to pay attention to such changes when patients are followed for several years after diagnosis.

## Materials and methods

### Ethical considerations

The project received approval from the Regional Committees for Medical and Health Research Ethics (REK) (reference number 245944), whereby REK granted an exemption from the consent requirement.

### Data

With pseudonymized health data from the national mandatory Cancer Registry of Norway, received on 01/02/2022, encompassing all cancer patients in Norway, we followed BC diagnosed patients, aged 40 plus at time of diagnosis, from 2006 throughout 2020. Patients were followed until breast cancer specific death (BCSD) or other causes of death (OCSD), until another BC event or end of study period. Cause of death was identified according to ICD-10 codes [8]. The time resolution was yearly, and patients who died within diagnosis year were given 0.5 years of survival. Patient characteristics were registered at time of diagnosis.

**Fig 1:**
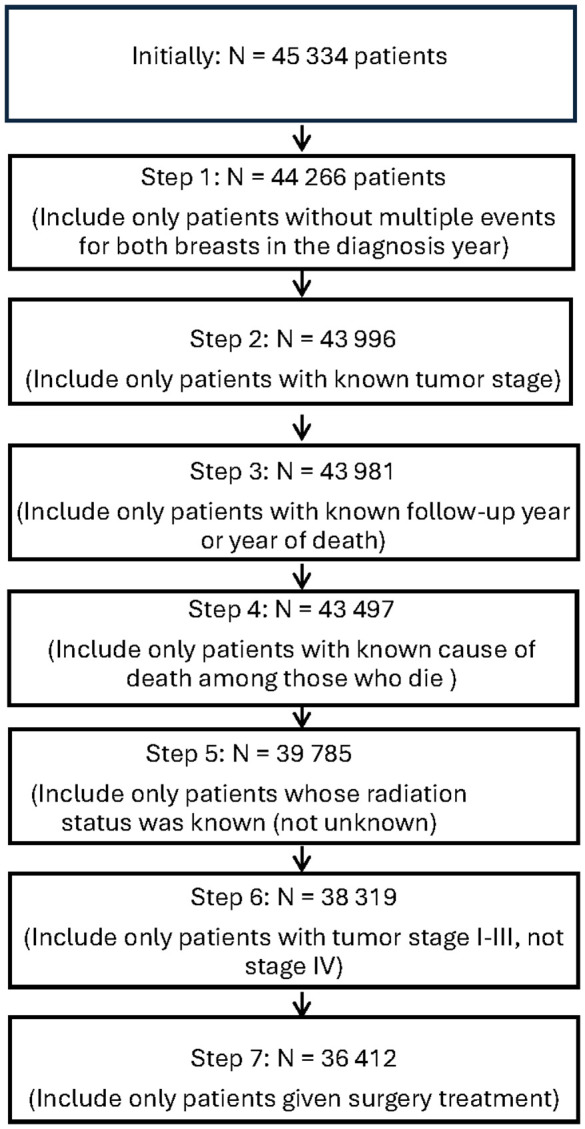
Flow diagram of the data selection procedure.

Data initially encompassed 45 334 patients, and after considering patients with valid timelines, one-sided BC event in the diagnosis year, known tumor stage I – III, surgery treatment, radiation status and cause of death, this reduced to 36 412 patients, see Fig 1. Note that a very large percentage of the patients had surgery treatment and very few were in tumor stage IV.

### Patient characteristics and treatment

BC treatment encompasses surgery, radiation, chemotherapy (cyclophosphamide, epirubicin, taxanes), trastuzumab, zoledronic acid and hormone therapy (anti-estrogen, tamoxifen, aromatase inhibitors), and usually patients receive a combination of treatments. Non-invasive and early-stage invasive BCs (stages I and II) have better prognoses compared to later stage BCs (stages III and IV). For patients in stage IV cancer has spread from the breast and nearby lymph nodes to other parts of the body, giving poor prognosis compared to earlier stages [9].

Treatment strategies depend upon patient’s molecular subtype and tumor stage, and treatment is hence associated with prognostic factors. We note that when analyzing register data one cannot assess how patient- and tumor-related prognostic factors are related to mortality in the absence of treatment, as in randomized trials. We hence do not examine the natural history of BC but rather describe survival for a large population given patient- and tumor-related prognostic factors and subsequent treatment for long term follow-up.

Overweight and obesity have been associated with poor prognosis and both overall and BC specific mortality, both for pre- and post-menopausal BC [10–12], partially mediated by tumor characteristics [13–14], although the literature is not unified [15]. Also, the magnitude of BMI’s effect on mortality for different BC patient subtypes is uncertain [16]. Jiralerspong and Goodwin stated findings to be generally consistent in the literature regarding decreased survival prognosis for obese HR positive patients, but less consistent for HR negative patients [14]. With information about BMI for 18 778 patients, we examined this, comparing BCSD for BMI patient groups, considering molecular subtype, age, tumor stage, and radiation therapy.

Patient’s BC categorizes into four molecular subtype groups:

1) hormone receptor (HR) positive and human epidermal growth factor receptor 2 (HER2) negative (HR + /HER2-),
2) HR positive and HER2 positive (HR + /HER2+),
3) HR negative and HER2 positive (HR-/HER2+) and
4) HR negative and HER2 negative (HR-/HER2-).

To be classified as HR + , the patient must either be estrogen receptor–positive or both estrogen receptor– and progesterone receptor–positive. Hormone therapy was not given to HR negative patients. HR and HER2 status are assessed according to national pathology guidelines aligned with ASCO/CAP recommendations [17,18]

Trastuzumab is generally offered to all eligible patients, with close monitoring for adverse effects and toxicity [19]. As explained in the introduction, these treatments together with chemotherapy are associated with prognostic factors, and we chose therefore not to include them in the analyses describing survival comparing patients with different molecular subtypes and BMI levels.

### Statistical analysis

As we are interested in BC ethology; the impact of having a molecular subtype or BMI level on BCSD, we consider the cause-specific hazard model approach, while an alternative approach, the Fine-Gray method, is better suited for prediction and not for answering etiological questions [20].

A popular model for survival is the Cox hazard regression model, requiring the so-called Cox proportional hazard assumption to be satisfied. This requirement is often not met, especially when analyzing long term survival data [21–22]. Then, often the alternative accelerated failure time (AFT) model is applied. But this latter approach does not solve the underlying problem of regression coefficients varying over time. Analyzing BC specific survival, the major interest lies in examining BCSD rather than OCOD. This competing risk situation, with OCOD as a competing risk, is especially expressed within an elderly population where the patients are at high risk of dying simply due to high age. We will explore cause-specific regression survival models for BC diagnosed patients and discuss challenges and alternatives for handling time dependent coefficients. Both the time varying nature of prognostic factors’ impact on BCSD and the high incidence of OCOD as a competing risk add challenges in describing and predicting long-term survival paths for BC patients.

We compared time to BCSD for patients with different molecular subtypes and patients in different BMI groups, given patient characteristics (age, tumor stage) and radiation therapy, assuming that all other treatments were given accordingly to guidelines.

We computed the overall incidence rates, the number of BCSDs and OCODs per patient year, and the corresponding rates for patients in the four molecular subtype groups. We further estimated empirically the cumulative incidence over time (nonparametric cumulative incidence functions and associated standard errors), expressing the probability of BCSD or OCOD for given groups of patients (defined by molecular subtype, tumor stage, BMI and age). We considered cause-specific Cox proportional hazard (PH) models, assuming constant hazard over time, i.e., that the relevant factors have a constant effect on the risk of death (hazard) over time. We examined this assumption and proposed two model alternatives allowing for shifting and time varying prognostic impacts of the relevant factors on BC mortality.

All analyses were done in the statistical program R, where we applied the survival package [23–24]. We used the ggcompetingrisks-function from the riskRegression package to plot the cumulative incidence functions (CIFs), which show the event-specific probability of BC death and other cause of death over time while accounting for competing risks [25]. We checked the Cox proportional hazard assumption by applying the cox.zph-function in R [26]. This function tests the proportionality of the factors entering the model by creating interactions with time. Statistical significance was evaluated at the 95% confidence level. Hazard ratios were considered significant at p < 0.05.

## Results

Patient summary statistics are given in Table 1. Patients were in one of the four molecular subtype groups. Altogether 63% of the patients were HR + /HER2-, while 7% were HR + /HER2 + . Similarly, 4% were HR-/HER2 + , and 27% were HR-/ HER2-. We extracted information on patients’ age, tumor stage and treatment (radiation therapy, chemotherapy (cyclophosphamide, epirubicin, taxanes), trastuzumab, zoledronic acid and hormone therapy (anti-estrogen, tamoxifen, aromatase inhibitors).

**Table 1:** Patient characteristics for all patients, patients who survive and patients who die due to BC or other causes (in %), for analysis comparing molecular subtypes (N = 36 412 patients).

	All (36 412)	Survived (30 745)	Death other causes (3 125)	Death due to breast cancer (2 542)
Molecular subtype 1-4^P^	63,7,4,27	67,7,4,23	47,5,2,46	38,6,6,51
Age group 1-4^A^	27,50,15,8	30,53,13,4	4,29,26,41	25,35,19,20
Tumor stage I-III^S^	51,38,11	55,36,10	43,46,10	18,51,31
Radiation therapy	78	82	41	66
Chemotherapy^C^	60	56	75	91
Trastuzumab	10	11	7	11
Zoledronic acid	73	70	96	75
Hormone therapy^H^	70	74	51	43

P: 1) HR + /HER2-, 2) HR + /HER2 + , 3) HR-/HER2+ and 4) HR-/HER2-

A: 1: 40–52, 2: 53–69, 3: 70–79, 4: 80 + years

S: clinical stage I (no tumor cells outside small tumor), clinical stage II (no tumor cells outside large tumor), clinical stage III (tumor cells within lymph glands around tumor)

C: cyclophosphamide, epirubicin or taxanes

H: anti-estrogen, tamoxifen or aromatase inhibitors

Altogether 2 542 patients, 7%, died from BC throughout the study period, and 8.6% died from other causes (3 125 patients). Among patients younger than 70 years, 3.7% and 5.5% died from other causes and BC, respectively. Among patients 70 years or older, on the other hand, 24.8% and 11.9% died from other causes and BC, respectively.

Information on BMI (through height and weight) was available for 18 778 patients. We categorized BMI as underweight (less than 18.5), normal weight (18.5–24.9), overweight (25–29.9) and obesity (30 or more) [27]. Summary statistics are given in Table 2. Altogether 2% were underweight, 44% normal weight, 35% overweight and 20% obese. Among patients experiencing BCSD 3% were underweight and 21% obese, while among patients experiencing OCOD the corresponding numbers were 7% and 14%, respectively.

**Table 2:** Patient characteristics for all patients, patients who survive and patients who die due to breast cancer or other causes (in %), for analysis comparing BMI grouped patients (N = 18 778 patients with registered height and weight (BMI)).

	All (18 778)	Survived (17 263)	Death other causes (757)	Death due to breast cancer (758)
BMI^B^	2,44,35,20	2,44,35,20	7,49,30,14	3,42,34,21
Molecular subtype 1-4^P^	76,8,4,12	78,8,4,10	73,7,3,17	51,7,8,35
Age group 1-4^A^	27,51,16,6	28,53,15,4	5,31,29,34	30,35,21,14
Tumor stage I-III^S^	52,35,13	54,34,12	44,44,13	12,41,47
Radiation therapy	84	86	46	74
Chemotherapy^C^	52	50	62	92
Trastuzumab	12	12	10	15
Zoledronic acid	73	72	95	70
Hormone therapy^H^	84	86	80	58

B: 1: < 18.5 (underweight), 2: 18.5–24.9 (normal weight), 3: 25–29.9 (overweight), 4: 30+ (obesity)

P: 1) HR + /HER2-, 2) HR + /HER2 + , 3) HR-/HER2+ and 4) HR-/HER2-

A: 1: 40–52, 2: 53–69, 3: 70–79, 4: 80 + years

S: clinical stage I (no tumor cells outside small tumor), clinical stage II (no tumor cells outside large tumor), clinical stage III (tumor cells within lymph glands around tumor)

C: cyclophosphamide, epirubicin or taxanes

H: anti-estrogen, tamoxifen or aromatase inhibitors

Table 3 displays overall incidence rates and incidence rates for patients in the molecular subtype groups, that is the ratio of the number of deaths to the total time at risk. All over, we see that the incidence rate for BCSD is somewhat higher than for OCOD. For patients with molecular subtype HR + /HER2- the incidence rates are similar (0.221 and 0.215 respectively). For the other three molecular subtype groups the BCSD incidence is higher than the OCOD incidence, especially for HR-/HER2+ and HR-/HER2- patients. If we consider 1000 patients in one year, we expect 221 of them to die from BC and 189 to die from any other cause. Similarly, we expect 306 and 245 patients with molecular subtype group HR-/HER2+ to die from BC and other causes, respectively.

**Table 3:** Number of events, person-years at risk, and incidence rates for everybody and for patients in the molecular subtype groups, for the events BCSD (breast cancer specific death) and OCOD (other cause of death).

		Event count	Person years at risk	Incidence rate (IR)
All patients	BCSD	2542	11525	0.221
	OCOD	3125	16494	0.189
Molecular subtype:				
HR + /HER2-	BCSD	963	4481.5	0.215
	OCOD	1464	6592.5	0.222
HR + /HER2+	BCSD	140	616.5	0.227
	OCOD	143	699.5	0.204
HR-/HER2+	BCSD	141	460.5	0.306
	OCOD	75	305.5	0.245
HR-/HER2-	BCSD	1298	5966.5	0.218
	OCOD	1443	8896.5	0.162

**Fig 2:**
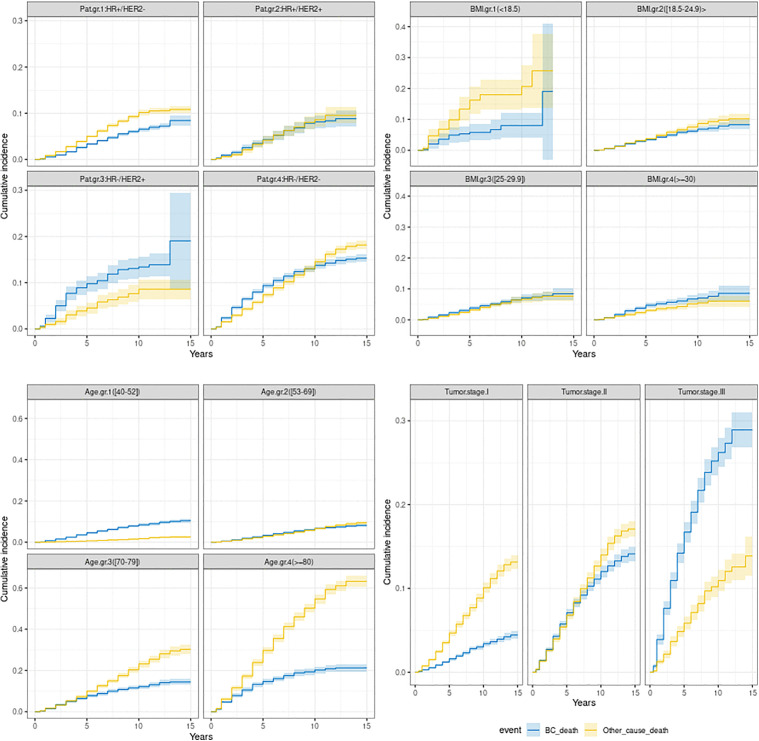
Cumulative incidences for patient-, BMI-, age- and tumor stage groups for patients who died due to breast cancer (blue lines) and due to other causes (yellow), with 95% confidence bands.

Now, in Table 3 we compute the overall incidence risk per patient year, but we are interested in how the incidence varies over time, and Fig 2 displays the cumulative incidence plots for BCSD and OCOD over time for the different molecular subtype-, BMI-, age- and tumor stage-groups.

We note differences in cumulative incidence among HR + /HER2- and HR + /HER2 + patients versus HR-/HER2+ and HR-/HER2- patients. There were relatively few patients in molecular subtype group 3 (HR-/HER2+) (see Table 1 and 2), especially being observed over several years, hence the wider confidence bands.

Underweight patients (BMI < 18.5) had both higher BCSD and OCOD cumulative incidence compared to patients with higher BMI, although we note that only 2% (out of 18 778 patients) were underweight. Also, we see that underweight patients had a higher cumulative incidence of OCOD compared to BCSD, while for patients in any other BMI group the cumulative incidence of OCOD was comparable to BCSD.

For patients in the youngest age group (40–52 years old at BC onset) the BCSD cumulative incidence was higher than the OCOD cumulative incidence. For patients 53–69 years old at BC onset the cumulative incidence of BCSD and OCOD were quite similar, while for older patients (70 years and older at BC onset) the cumulative incidence of OCOD was (much) higher than that of BCSD, and this difference increased over time. The latter is of course not surprising as patients 70 years or older at BC onset have a higher mortality rate due to just being old, and this difference also obviously increases as time goes by.

The OCOD cumulative incidence was quite similar across all tumor stages, while the cumulative incidence of BCSD increased rapidly over time with an increasing tumor stage, from just under 0.5% for patients in tumor stage I after 15 years, to just under 30% for patients in tumor stage III after 15 years.

**Fig 3:**
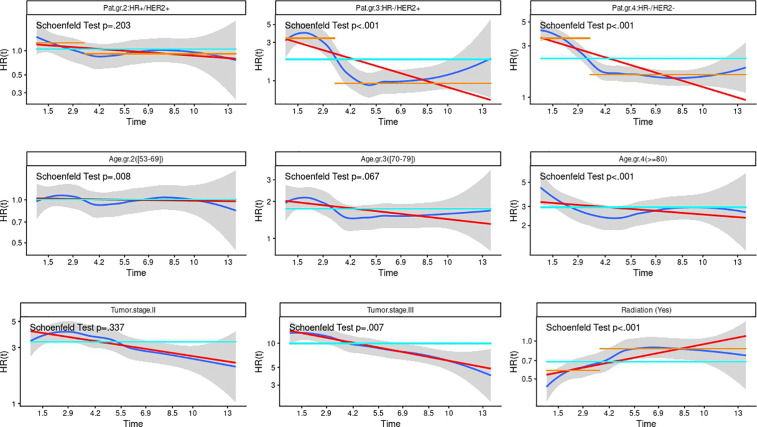
Estimated hazard ratios over time for breast cancer specific death for factors entering the model (baseline: molecular subtype HR + /HER2-, age 40−52 years, tumor stage I, no radiation therapy). The blue line is the hazard ratio fitted by a smoothing spline over time, and the gray area shows a + /- 2-standard-error band around this blue line. The turquoise line is the estimated time invariant hazard rate (HR) in a Cox-model when we ignore the possibility of time varying coefficients. For each level of the factors, we compute the Schoenfeld residuals over time, and the p-value obtained from the Schoenfeld test for each level of the factors is displayed.

We first fitted a Cox model for BCSD, with OCOD as a competing risk and the results are given in Table 4. We checked the Cox proportional hazard assumption and in Fig 3 we plotted the results from this test; the estimated hazard ratios over time, where the blue line is the hazard ratio fitted by a smoothing spline varying over time, and the gray area shows a + /- 2-standard-error band around this blue line. The turquoise line is the estimated time invariant hazard rate (HR) in a Cox-model when we ignore the possibility of time varying coefficients, as seen in Table 4. For each level of the factors entering the model, we computed the Schoenfeld residuals over time [26,28]. We tested for independence between these residuals and time, and small p-values indicate time dependence, i.e., a violation of the proportionality assumption.

**Table 4:** Hazard ratio estimates for BCSD, with OCOD as a competing risk. Results are considered significant at p < 0.05.

Cox model with time invariant regression coefficients				
Factor	Level	Hazard ratio	95% CI	P-value
Molecular subtype	HR + /HER2 + /HER2-	11.00		
	HR + /HER2+	1.04	(0.87,1.24)	0.655
	HR-/HER2+	1.85	(1.54,2.21)	<0.001
	HR-/HER2-	2.29	(2.10,2.49)	<0.001
Age group	40-52	1.00		
	53-69	1.01	(0.91,1.12)	0.898
	70-79	1.75	(1.55,1.97)	<0.001
	80+	2.95	(2.59,3.35)	<0.001
Tumor stage	I	1.00		
	II	3.36	(3.02,3.74)	<0.001
	III	9.93	(8.81,11.20)	<0.001
Radiation therapy	No	1.00		
	Yes	0.68	(0.62,0.75)	<0.001
Cox model with time dependent regression coefficients for molecular subtype groups and radiation treatment*				
Factor	Level	HR	95% CI	P-value
Molecular subtype, time <=3 years	HR + /HER2	1.00		
	HR + /HER2+	1.25	(0.95,1.64)	0.111
	HR-/HER2+	3.38	(2.70,4.23)	<0.001
	HR-/HER2-	3.53	(3.10,4.02)	<0.001
Molecular subtype addition after 3 years^†^	HR + /HER2	1.00		
	HR + /HER2+	0.73	(0.51,1.05)	0.087
	HR-/HER2+	0.28	(0.19,0.40)	<0.001
	HR-/HER2-	0.46	(0.39,0.55)	<0.001
		1.00		
Age group	53-69	1.01	(0.91,1.11)	0.908
	70-79	1.76	(1.56,1.98)	<0.001
	80+	2.89	(2.53,3.28)	<0.001
Tumor stage	I	1.00		
	II	3.35	(3.01,3.73)	<0.001
	III	9.84	(8.73,11.09)	<0.001
Radiation therapy, time <=4 years	No	1.00		
	Yes	0.59	Age group	40-52
Radiation therapy addition after 4 years^†^	No	1.00		
	Yes	1.49	(1.26,1.77)	<0.001

† Hazard ratio for molecular subtype group HR-/HER2- versus HR + /HER2- (baseline) after more than 3 years following BC onset is given by 3.53*0.46 = 1.62

*The breakpoints of 3 years (molecular subtype) and 4 years (radiation therapy) were found from a fitted model allowing for smoothing splines over time

We see from Fig 3 that the Cox proportional hazard assumption was not met with many of the factor levels. For patients 53–69 years old we see that the gray area covers one, indicating no difference in risk of BCSD among these patients and those in the baseline group (40–52 years old), as also seen in Table 4. For patients 70 years and older (age groups 3 and 4) we see that hazard ratios were somewhat higher in the first years, and then flattened out, but it was clearly higher than one at any time. The hazard ratios for patients being in tumor stage II and III versus tumor stage I were clearly much higher than one at any time point. Assuming a constant hazard ratio slightly underestimates the hazard early on and overestimates it later. The Schoenfeld test results stated that for some levels of the factors age and tumor stage the time invariance requirement was met and for some levels it was not. Given that the hazard ratio is either close to one through the years (age group 2 versus 1) or far from one (tumor stage III versus stage I) or that the Schoenfeld test p-value was greater 0.05 we propose a model with time-invariant coefficients these factors.

For HR-/HER2+ and HR-/HER2- patients we see a shift in BCSD risk around 3 years after BC onset, compared to HR + /HER2- patients (the baseline group). Similarly, for patients given radiation therapy versus no radiation therapy we see shift in BCSD risk R around 4 years after BC onset. We therefore suggest a model with time dependent coefficients for molecular subtype groups and radiation therapy as shown by the orange lines in Fig 3. This implies that the model estimates one hazard ratio for each of the molecular subtype groups with HR + /HER2- patients as the baseline for the first 3 years following BC diagnosis and another hazard ratio for the subsequent years. Similarly, we allowed for different hazard ratios for patients treated with radiation therapy with no treatment as baseline the first four years following BC diagnosis and the subsequent years. The resulting fitted Cox model is also displayed in Table 4.

We examined models allowing for interaction between molecular subtype and age and molecular subtype and tumor stage, but these interaction terms were not significant.

In a traditional Cox model, ignoring the possibility of time varying coefficients, we found no significant difference among HR + /HER2- (baseline group) and HR + /HER2 + patients, while HR-/HER2+ and HR-/HER2- patients have both clearly higher HR. Patients given radiation therapy had a decreased risk for BCSD following BC diagnosis with a hazard ratio of 0.68 (95% CI:0.62–0.75).

In the model allowing for time varying coefficients for molecular subtype groups and radiation therapy, we found that HR-/HER2+ and HR-/HER2- patients had hazard ratios of 3.38 (95% CI: 2.70–4.23) and 3.53 (95% CI: 3.10–4.02) compared to HR + /HER2- (baseline group) within 3 years following BC diagnosis. The corresponding numbers in subsequent years were 0.931 and 1.62, respectively. Hence, after 3 years following BC diagnosis, we see no difference in BCSD among HR-/HER2+ and HR + /HER2- patients. The positive effect of radiation therapy on BCSD was clearly highest in the first years following diagnosis, but still positive, albeit much smaller, in the later years (HR = 0.87).

In the analysis focusing on patients with different BMI-levels, we found no difference in risk of BCSD between patients underweight, normal weight, overweight and obese when adjusting for molecular subtype, age, tumor stage and radiation therapy, and if each patient received chemotherapy, trastuzumab, zoledronic acid and hormone treatment according to guidelines. In a preliminary analysis where we included the patients without surgery treatment and of course also adjusting for this, we found that underweight patients had an increased risk for BC specific death compared to normal weight patients. The few underweight patients without surgical treatment had relatively shorter survival time compared to normal weight, overweight and obese patients also without surgical treatment.

We examined models allowing for interaction between BMI and molecular subtype, BMI and age and BMI and tumor stage, but these interaction terms were not significant.

## Discussion

We analyzed time to BCSD by a Cox model with OCOD as a competing risk, where we focused on molecular subtype and BMI patient groups, adjusting for age, tumor stage and radiation therapy, assuming other treatments were given according to guidelines. We found violations of the Cox proportional hazard assumption and showed that it was sensible to let the coefficients for the molecular subtype groups and for radiation therapy be time dependent.

In our analysis we found no significant difference in BCSD hazard between HR + /HER2- and HR + /HER2 + patients at any time following BC onset. Considering HR-/HER2+ and HR-/HER2- patients we found that for the first three years following BC diagnosis these patients had hazard ratios of 3.38 (95% CI: 2.70–4.23) and 3.53 (95% CI:3.10–4.02) compared to HR + /HER2- patients, respectively. Hence, HR negative patients are expected to have higher risk of BCSD than HR positive patients in the first 3 years following BC onset. This corresponds to a previous publication, reporting almost twice as high overall mortality for HR negative compared to HR positive patients [29]. Also, Dunnwald et al reported lower mortality risk among HR positive patients versus HR negative patient groups, regardless of age [30]. But we also found that after 3 years this increased BCSD risk declined and for the HR-/HER2 + group it was comparable to that of HR + /HER2- patients. HR- patients lack estrogen and progesterone receptors, and such patients are more prone to early recurrence and hence increased BCSD risk the first years, but later this risk decreases. This is comparable to what Pan et al reported, that HR negative tumors had poorer prognosis the first five years, but not afterwards [31].

BC specific mortality was higher with increasing age and tumor stage. Radiation treatment reduces the risk of BC recurrence, and this is known as a major factor that influences BC survival in the early years following the treatment. When doctors consider patients for radiation treatment, they weigh the benefits of preventing BC recurrence against potential side effects. We found patients to have benefited from radiation treatment with respect to BCSD in the first years. But after about four years we found these patients to have a risk of BCSD no different from those who were not given radiation treatment. This was in line with what Dubois et al found at 5-year BC survival [32]. Kunkler et al reported in a study that radiation treated patients benefited from this treatment compared to the non-treated patients, but the two groups of patients obtained about the same overall survival 10 years later [33]. Comparing this to our analysis, we found in Fig 3 studying the impact of radiation treatment a hazard ratio closest to 1 for the nonlinear blue curve at approximately 7 years, that is 3 years earlier. The study of Kunkler et al included 1326 patients 65 years and older who were HR+ in tumor stage I or II, corresponding to a substantial fraction of our patients (see Table 1) [33].

Trastuzumab is given HER2 positive patients as targeted treatment since this genetic mutation is linked to poorer prognosis [34]. Patel et al. summarized that trastuzumab can prolong HER2 positive patients’ survival [35]. We found that HR + /HER2 + patients (given trastuzumab) and HR + /HER2- patients (not given trastuzumab) did not have significantly different BC specific mortality. This can indicate that receiving trastuzumab treatment could compensate for the disadvantage of having a HER2 positive diagnosis compared to a HER2 negative diagnosis with respect to BCSD.

We found no difference with respect to the risk of BCSD among patients with different BMI. The literature regarding BMI and survival is not unified, and Moon et al. stated that such an association is controversial [36]. Jiralerspong and Goodwin concluded that obesity was associated with poorer, both general and BC specific, survival [14]. Such differences were not significant in our analysis. On the other hand, Saleh et al. concluded that low BMI was associated with better survival while overweight and obesity was not [15]. Allover, literature diverges on this topic, and several papers have discussed what is called the “obesity paradox”. This is when patients with higher BMI could experience better outcomes than patients with normal BMI. Lennon et al list several methodological and clinical explanations [37]. They refer to examples where tumors among obese patients had less aggressive characteristics compared with those among patients with normal weight. They also pointed out that higher BMI patients had metabolic reserves that could aid in combating diseases and tolerating treatments such as chemotherapy. The “obesity paradox” could be due to a combination of many factors such as metabolic reserves, differences in chemotherapy dosing, hormonal interactions, and possible explanations that stems from study design features, and is yet to be fully understood. Some of the many driving factors and their complicated interactions could perhaps have “cancel each other out” each other in the regression analysis and thus explain our results of no significant differences in BC survival among BMI patient groups. Also, as others also have pointed out, typically we only know BMI at time of BC initiation or from the initial treatment period, and this limits the discussion. We believe future studies should pay attention to the impact of BMI on BCSD considering the patient’s treatment trajectory and its effect over time.

The Cox modelling approach is popular, but the underlying non-proportionality assumption is not always checked. Altman et al. found in their review most publications on cancer survival did not do an assumption check, although we believe this has improved since then [38]. As Bellera et al. points out, using the Cox model wrongly could give misleading results where for example the estimated coefficient underestimates the first time period under consideration and overestimates later, or vice versa [18]. This was clearly the case here, where a model with time invariant coefficients did not disclose the decrease in BCSD risk in the later years for HR-/HER2+ and HR-/HER2- patients versus HR + /HER2- patients as seen in Fig 2. The same was true for radiation therapy versus no such therapy, where we see a much higher reduction in BCSD risk the first versus the latter years. Within BC research and cancer research in general, five-year survival, incidence, and mortality are important metrics for surveillance and comparison. This seems understandable, given the time varying nature of the coefficients in Fig 2, although we see a change in several of the coefficients earlier than five years following diagnosis.

Now, looking at the hazard ratio fitted smoothing splines (blue lines) and gray confidence bands in Fig 2 for the HR-/HER2+ and HR-/HER2- patients, we see that an alternative model would be to allow for one risk level for time up to about 2.5 years following diagnosis, then to let the risk be a linear function of time until 5 years following diagnosis and later on assume one common risk level. Similarly, for describing the BCSD risk for patients given radiation therapy or not, we could have considered the risk to be a linear function of time until 5.5 years and then one risk level for the time afterwards. This would have been a more detailed, but also more complicated model than the one suggested. In designing useful statistical models there is a delicate balance between complexity, interpretability, and generalization of the models.

We lastly note that if we had been interested in absolute risk prediction and not in understanding the biological or etiological effect of the covariates on a specific BCSD death, and hence applied a Fine Gray modelling approach, the challenges of time varying coefficients would be the same.

## Limitations

Because our analysis was based on a population-wide dataset, the potential of selection or observational bias was minimized. Data on everybody diagnosed with BC in Norway are contained and followed up in the Cancer Registry of Norway.

The exact date of BC diagnosis was not available and hence prevented a precise calculation of survival time. Information on radiotherapy and other treatments was limited to whether treatment was given or not, without information on treatment timing. Consequently, e.g., radiotherapy status may reflect post-baseline information. We acknowledge that this may introduce immortal time bias, as patients classified as having received radiotherapy and other treatments must have survived long enough to receive treatment. Still, we note that Norwegian breast cancer care is standardized, with limited expected variation in time to treatment.

Knowledge of cause of death was required to distinguish breast cancer‑specific mortality from other causes, and we therefore restricted the analysis to patients with known cause of death, excluding a small proportion of patients. We acknowledge that this cohort restriction may introduce selection bias if patients with unknown cause of death differ systematically from those with known cause of death. However, given the small proportion of excluded patients, we consider the risk of substantial bias to be limited.

The lack of information on prognostic factors such as tumor grade, reflecting biological aggressiveness rather than disease extent, and comorbidity represents a limitation of this study and may have influenced the results through residual confounding. There was no information on treatment doses or other treatment details. Therefore, a detailed analysis incorporating such data was not possible.

As systemic treatments are largely determined by molecular subtype and administered after diagnosis, they were considered potential mediators rather than confounders and were therefore not adjusted for. This was considered reasonable, as the aim was to estimate the overall association between molecular subtypes and mortality.

## Conclusions

Our findings contribute to understanding long-term survival among BC patient groups, with respect to molecular subtype and BMI, age, tumor stage and treatment. We have shown known factors’ time varying impact on BCSD and especially suggested how to specify a simple model allowing for time varying coefficients for molecular subtype groups and radiation therapy.
